# Discover your secret sauce – an interview with Chien-Yu Chen on career choices and machine learning

**DOI:** 10.1038/s42003-022-03080-x

**Published:** 2022-02-17

## Abstract

Dr Chien-Yu Chen is a Professor at the Department of Biomechatronics Engineering at National Taiwan University (NTU) in Taipei. She received a PhD degree in Computer Science and Information Engineering from NTU and has been leading her lab since 2005. Dr Chen develops machine learning and deep learning models to study multi-omics data, including genomes, transcriptomes, epigenomes, and proteomes.

**Figure:**
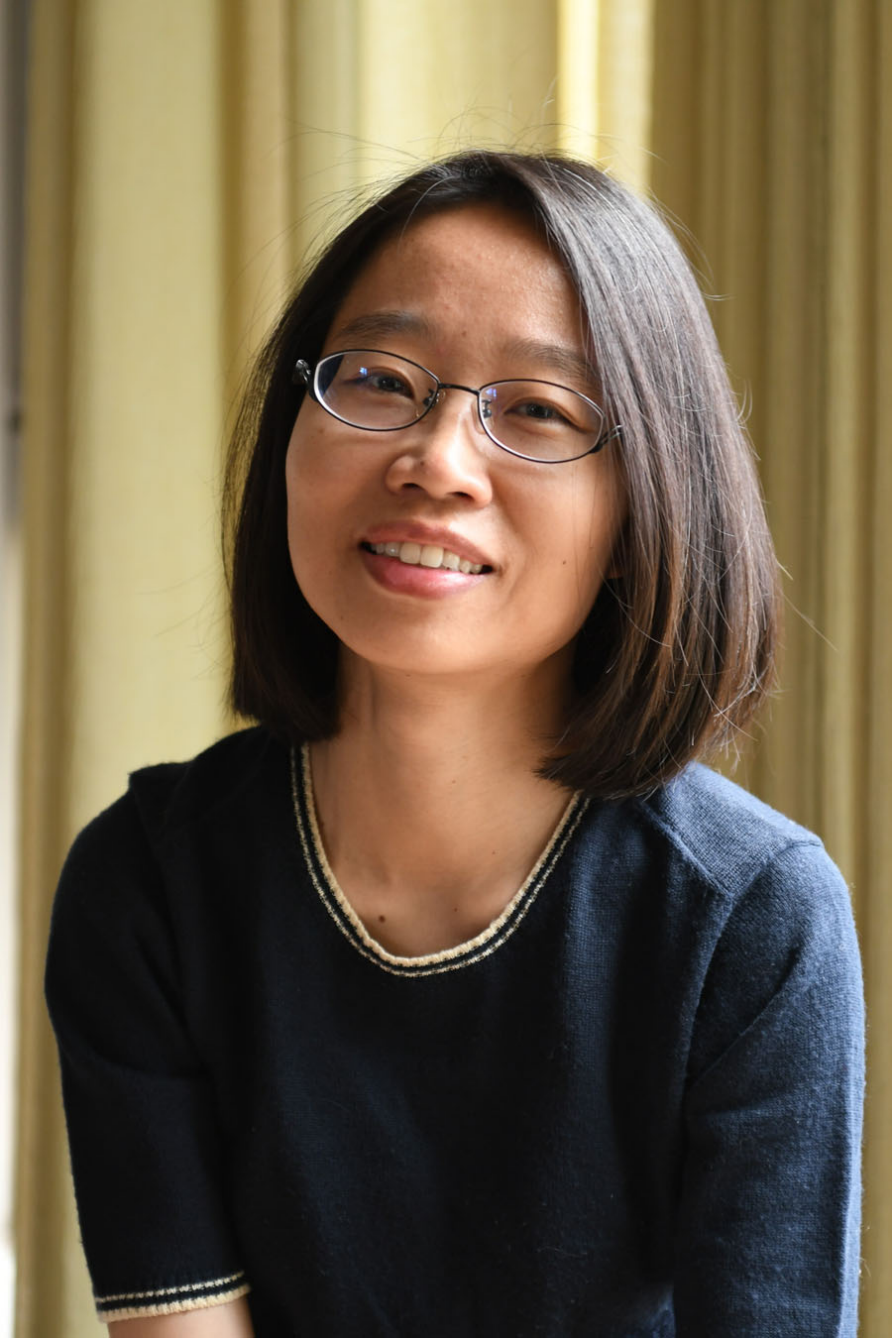
Chien-Yu Chen

Tell us about your research interests!

My research interests focus on developing computational methods for studying gene regulation and immunogenomics. The proposed methods usually involve components of machine learning or deep learning. For examples, recently we developed an AutoML package, ezGeno, for analyzing epigenomics data. Also, the manuscript just published in *Communications Biology* presented a deep learning approach to predict and summarize MHC-I binding peptides with respect to different HLA alleles. These two examples explain what kind of scientific questions inspire me and how I and my team leverage machine learning or deep learning techniques to tackle these questions.

You studied to be an electrical engineer. What made you move into computer science?

My first job after receiving my master’s degree in electrical engineering was as an integrated circuit (IC) design engineer. The job entailed implementing digital circuits for pre-defined specification. When I was working in an IC design house, I noticed that hardware design engineers largely utilize software to accelerate IC design cycles. This motivated me to tap into computer science. Within a short period of time in the first job, I realized that being an engineer cannot and will not satisfy me. As the days went by, it gradually became clear to me that I always enjoyed learning new things and sharing what I have learned with other people. This naturally led to my decision to pursue a Ph.D. degree in computer science in 1999 and to be an educator later.

Women are still underrepresented in computer science. What advice would you give to women thinking about a career in this field?

I think the key point is to ask yourself whether you truly like the subjects in computer science, no matter whether you are male or female. If the answer is yes, then have fun - you have got everything you need to enjoy the journey of studying computer science. For example, I noticed that I am good at logical thinking. When studying electrical engineering, I really liked logic design and computer architecture which is heavily tilted toward the logical thinking processes. Maybe I just happen to be good at understanding things in abstract representation. When studying computer science, I really enjoyed studying algorithms and computation theory. I admire people who create such elegant algorithms that solve difficult problems efficiently. This is really amazing. My proclivity toward logic thinking has been my secret sauce. My advice is to find your own secret sauce and let it empower you and lead you through your career just like how mine empowered me and led me to the academic path.

There has been remarkable progress in your field in recent years. How do you see the future for machine learning in biology and/or medicine?

I believe machine learning (including deep learning) will revolutionize biology and medicine on a large scale. One of the major objectives of biomedical studies is to prevent or eliminate human diseases. The property of machine learning is learning from data, especially from a large amount of data. I expect machines to learn useful skills even on the tasks that an expert cannot do well. We know that biological systems are built based on the DNA blueprint. With the human genome assembly completed and released in 2001, in theory, we have the chance to understand humans by learning how to interpret the DNA language. However, deciphering the DNA language is not easy. The biological systems are so complicated, especially when variants on the genome cause diseases such as cancers or inherited diseases. In these situations, the causal-effect relationship between variants and diseases are so complex that cannot be solved by linear models. Yet, it may be solved by deep neural networks provided that there is a large amount of disease-associated genetic variant data. By the advance of novel biotechnologies that generate abundant data, machine learning has the role to help understand the biological systems by learning from data. I am optimistic and curious to see how this will drive medicine to improve in the future.

What are the main challenges?

We need more data with good quality and labeling.

Your recent paper in *Communications Biology* was submitted through the “Guided Open Access” initiative, in which editors at 3 journals collaborate to evaluate the manuscript.

How was your experience with this pilot?

Actually, I came across GOA by accident. But I really enjoyed the service during the period of manuscript submission and revision. The editors and reviewers gave us very useful advices that helped us to improve the manuscript. And I am glad that our paper was published under such an efficient and expert guidance.

What do you feel is the value of publishing Open Access?

No doubt, the visibility of a study is largely increased by Open Access publication. It really helps our research to reach a broader audience.

*This interview was conducted by Deputy Editor Christina Karlsson Rosenthal*

